# Eculizumab as a New Treatment for Severe Acute Post-infectious Glomerulonephritis: Two Case Reports

**DOI:** 10.3389/fmed.2021.663258

**Published:** 2021-07-26

**Authors:** Hassib Chehade, Gabriella Guzzo, Francois Cachat, Samuel Rotman, Daniel Teta, Giuseppe Pantaleo, Salima Sadallah, Amita Sharma, Ivy A. Rosales, Nina Tolkoff-Rubin, Manuel Pascual

**Affiliations:** ^1^Department of Paediatrics, Lausanne University Hospital, University of Lausanne, Lausanne, Switzerland; ^2^Transplantation Centre, Lausanne University Hospital, University of Lausanne, Lausanne, Switzerland; ^3^Department of Immunology and Allergy, Lausanne University Hospital, University of Lausanne, Lausanne, Switzerland; ^4^Division of Nephrology, Valais Hospital, Sion, Switzerland; ^5^Department of Clinical Pathology, Lausanne University Hospital, University of Lausanne, Lausanne, Switzerland; ^6^Division of Nephrology, Massachusetts General Hospital, Boston, MA, United States; ^7^Division of Pathology, Massachusetts General Hospital, Boston, MA, United States

**Keywords:** post-infectious glomerulonephritis, terminal complement pathway blockage, anti-c5 monoclonal antibody, eculizumab, case report

## Abstract

Acute post-infections glomerulonephritis (APIGN) is a frequent cause of glomerulonephritis and represents the most common cause of acute glomerulonephritis in children. It can evolve to severe acute renal failure and chronic kidney disease or even end-stage kidney disease. The precise pathophysiological mechanisms of APIGN are still incompletely understood. The implication of the alternative complement pathway and the potential benefits of C5 blockade have been recently highlighted, in particular in the presence of a C3 Nephritic Factor (C3Nef), anti-Factor B or H autoantibodies. We report two children with severe APIGN, successfully treated with eculizumab. The first patient presented a severe form of APIGN with advanced renal failure and anuria, associated with a decreased level of C3 and an increased level of soluble C5b-9, in the presence of a C3NeF autoantibody. The second case had a severe oliguric APIGN associated with low C3 level. Kidney biopsy confirmed the diagnosis of APIGN in both cases. Eculizumab allowed full renal function recovery and the avoidance of dialysis in both cases. In conclusion, the alternative and terminal complement pathways activation might be common in PIGN, and in severe cases, eculizumab might help.

## Introduction

Acute post-infectious glomerulonephritis (APIGN) represents the major cause of acute glomerulonephritis in children worldwide. It is often secondary to group A streptococcal infection (1). In 2005, the World Health Organization estimated the number of new annual cases at 472,000, with a mortality of 5,000 cases per year (2). The vast majority of cases occurs in developing countries with an annual incidence of 24/100,000 inhabitants (2). Although APIGN harbors a good prognosis, it is rarely associated to permanent mild urinalysis abnormalities, acute renal failure, or even end-stage kidney disease (3–5).

The efficacy of immunosuppressive agents in APIGN has not been demonstrated and conservative care often remains the sole management strategy (1, 3, 6, 7). However, the treatment of severe forms of APIGN may nowadays improve based on the availability of effective complement inhibitors. Complement activation has indeed been shown to be instrumental in the pathophysiology of APIGN, and several reports have highlighted a major role of terminal complement activation in the development of post-infectious glomerular injuries (8–10). We have recently reported the first case of successful reversal of acute and severe PIGN using eculizumab, an anti-C5 monoclonal antibody that blocks terminal complement activation, in an 8-year-old child with APIGN requiring dialysis (11).

We hereby report the successful terminal complement blockade using eculizumab in two children with severe crescentic APIGN. These observations further emphasize the critical role of terminal complement activation in APIGN glomerular inflammation and injury and, most importantly, they support the strategy of blocking terminal complement activation with an anti-C5 monoclonal antibody in patients with severe APIGN.

## Case Description

### Case 1

A 6-year-old boy, with no prior medical history, was admitted to the University Hospital of Lausanne with gross hematuria, abdominal pain, and oliguria. He reported a pharyngitis 7 days earlier. On physical exam, there was no edema, and cardiovascular, lung, neurological, and skin examinations were all unremarkable. Blood pressure was 120/90 mmHg. Urinalysis displayed numerous red blood cells casts and dysmorphic red blood cells, and proteinuria (urine protein/creatinine ratio 1,300 g/mol). Complete blood count showed a normocytic non-hemolytic anemia (Hb 97 g/L, normal white blood cells and platelets). Serum creatinine and BUN levels were at 46 and 14 mmol/L, respectively. Complement measurements showed low plasma complement C3 (0.27 g/L; norm: 0.75-1.4 g/L), C4 (0.08 g/L; norm: 0.10-0.34 g/L), and CH50 (29%; norm: 70-140%). Circulating C5b-9 was at 2,547 ng/ml (norm: 130-350 ng/ml). The C3 levels were assessed by nephelometry (BNP Prospec, Siemens Behring) with reference ranges supplied by the vendor (normal range 75–175 mg/dl). Plasma sC5b-9 level was measured with an ELISA kit developed by Quidel: MicroVue sC5b-9 Plus EIA kit. No anti-Factor B and H autoantibodies were detected, but C3 Nephritic Factor (C3NeF) was present. Anti-nuclear antibody and anti-neutrophil cytoplasmic antibodies were undetectable. Antistreptolysin O were at 740 UI/ml (norm: <250 UI/ml).

**Figure 1:**
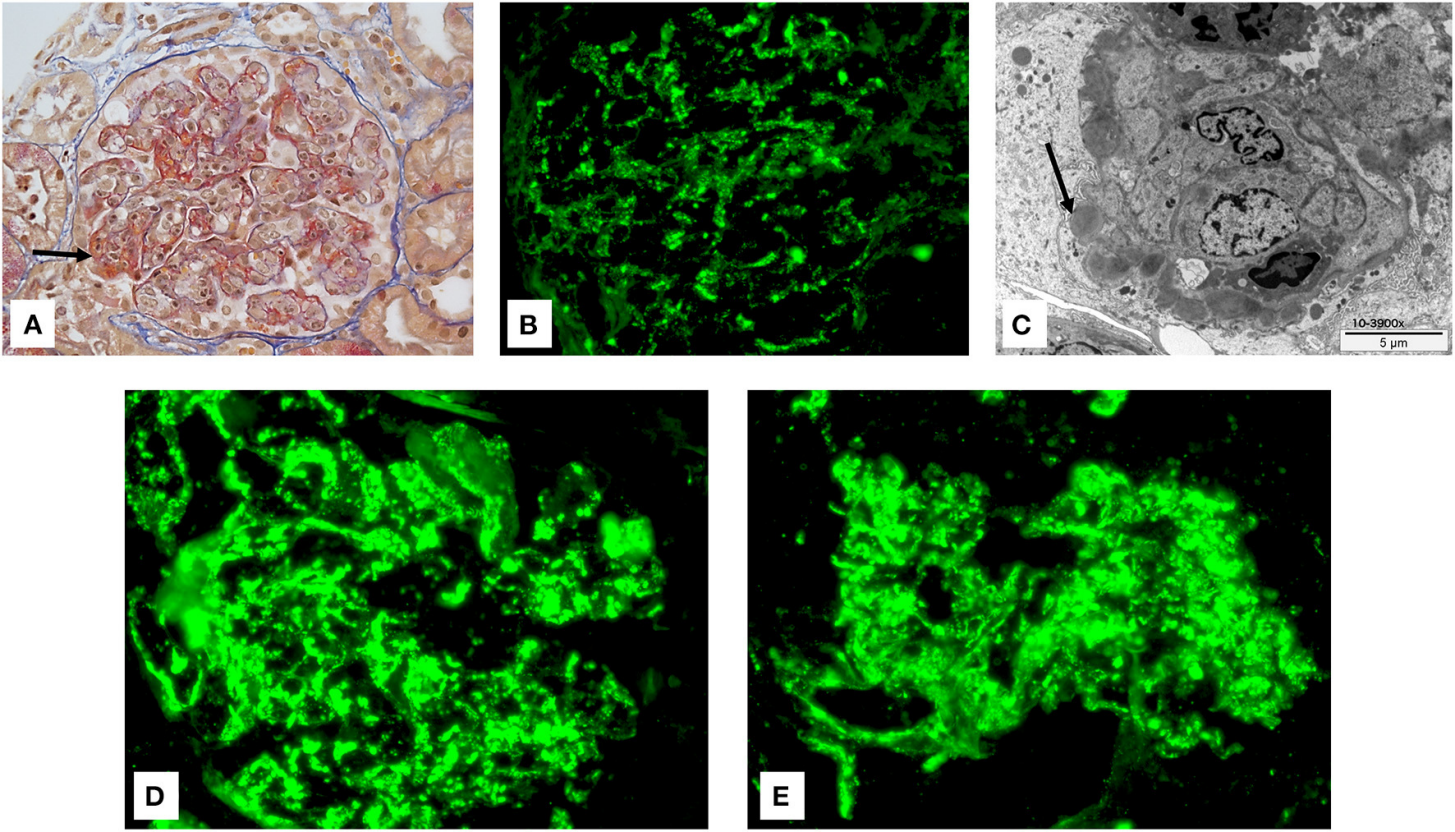
**(A)** Glomeruli show a lobular flocculus with a global and diffuse endocapillary proliferation associated with mesangial hypercellularity. Crescents with fibrinoid necrosis were present (arrow) (Trichrome stain: original magnification: 400×). **(B)** By immunofluorescence, IgG deposits were diffusely observed and organized in a starry sky pattern (FITC: original magnification: 400×). **(C)** Numerous subepithelial (humps) and mesangial dense deposits were observed by electronic microscopy. Arrow shows so called humps. **(D,E)** By immunofluorescence, C3 **(D)** and C5-9 **(E)** deposits were diffusely observed (FITC: original magnification: 400×).

Twelve hours after admission, the child developed anuria, and his serum creatinine and urea nitrogen levels further increased to 347 and 26.1 mmol/L, respectively, associated with hyperkalemia (5.6 mmol/L), hyponatremia (128 mmol/l), hyperphosphatemia (2.97 mmol/l), and metabolic acidosis (bicarbonate 19 mmol/l). Renal biopsy showed an exudative diffuse glomerulonephritis with enlarged and hypercellular glomerular tufts. Mesangial and endocapillary hypercellularity were present, with crescent formation in 2 out of 21 glomeruli (Figure 1A). Immunofluorescence revealed abundant IgG, C3 and C5-9 deposits arranged in starry sky pattern (Figures 1B,D,E). Ultrastructural analysis displayed subepithelial electron-dense “humps” (Figure 1C). In view of the renal biopsy findings with extensive C5b-9 deposits, and of the severity of the PIGN, a single dose of intravenous eculizumab (600 mg) was administered with parental consent. The child had already received meningococcal immunization with a tetravalent (A, C, W, Y) conjugated vaccine. Daily antibiotic prophylaxis with penicillin V was administered for a total of 6 weeks. Response was immediate, with brisk diuresis starting 12 h after eculizumab administration. Serum creatinine level normalized in 48 h, and proteinuria decreased dramatically and normalized after 2 months. Circulating sC5b-9 levels decreased from 2547 ng/ml at day 0 to <130 ng/ml at day 20, whereas plasma complement C3 normalized at day 28. C3NeF was undetectable at day 20. Genetic analyses of the alternative pathway complement proteins were normal.

Two years later, the child is doing well with normal blood pressure, normal renal function, normal complement levels, undetectable C3NeF, normal urinalysis, and no proteinuria.

### Case 2

A previously healthy 8-year-old girl presented to an outside hospital with a one-day history of sudden onset gross hematuria and flank pain. Serum creatinine was 106 μmol/L on presentation and non-contrast CT scan of the abdomen was unremarkable. There was no preceding pharyngitis, rash, arthritis, skin infection, fever, or diarrhea. Vital signs were: BP 111/57 mmHg, heart rate 60 beats/min, respiratory rate of 20/min. Physical exam was normal. Because of rapidly worsening serum creatinine (up to 246 μmol/L), decreasing urine output (<1 cc/kg/h) and edema, she was transferred to the Massachusetts General Hospital in Boston (USA) for further management. At this time, laboratory work up showed a decreased complement C3 (46 mg/dl, norm: 93-202 mg/dl), normal complement C4 (27 mg/dl, norm: 13-51 mg/dl) with total complement <41 units/ml (norm: 63-145 units/ml). Antinuclear antibodies, anti-dsDNA, anti-neutrophil cytoplasmic antibodies and anti-glomerular basement membrane antibody were negative. Antistreptolysin O titer was 683 IU/ml (norm: 0-375 IU/ml). A kidney biopsy obtained on day 2 showed 32 glomeruli, with no significant sclerosis. Cellular crescents were present in four glomeruli. All glomeruli showed endocapillary hypercellularity with intracapillary neutrophils. There was minimal mesangial hypercellularity. Immunofluorescence showed prominent mesangial staining for C3 with focal segmental granular glomerular basement membrane staining for IgG. Electron microscopy showed many mesangial electron-dense deposits and subepithelial humps. These findings were consistent with an APIGN with crescents and C3 predominance.

The patient was started on intravenous methylprednisolone 500 mg twice a day for 2 days. However, she continued to be oliguric, hypertensive and edematous. Serum creatinine peaked at 563 μmol/L on day 3. A single dose of eculizumab (1,200 mg) was then administered after formal parental informed consent was obtained. She was vaccinated against meningococcal disease and was given Ciprofloxacin prophylaxis.

The urine became clear within 2 h after infusion. Urine output increased significantly within 24 h. Serum creatinine normalized after 2 weeks and urine sediment normalized in 3 months. Serum C3 returned to normal level after 4 weeks. A second eculizumab dose (900 mg) was administered 15 days after the first dose. There were no mutations detected in Complement Factor H, Complement Factor I or membrane cofactor protein and further tests show normal alleles of Complement Factor H-related 5. C3NeF was normal. Renal function remains preserved for 2 years now, with normal complement levels and urinalysis.

## Discussion

APIGN is an immune-mediated glomerulonephritis complicating a non-renal bacterial infection. The time lapse between infection and the onset of APIGN complication suggests a pathogenic role for preformed or glomerular-planted immune-complexes mediated by the infectious stimuli (12, 13). In several cases of APIGN, immune-complexes may activate the classical complement pathway, leading to C4 consumption, and IgG deposition in glomeruli (14, 15). However, low C3 level is observed in almost 90% of biopsy-proven APIGN, whereas levels of the classical complement activation pathway such as C1q, C2, and C4, are normal or only slightly depressed (16, 17). In addition, properdin and C3 deposition may precede IgG deposition, or may be present without IgG (18–20). In an *in vitro* model, Yoshizawa et al. observed that streptococcal protein, such as the nephritis-associated plasmin receptor induces C3 activation *via* the alternative complement pathway (21). Recently, Chauvet et al. identified autoantibodies against factor B in 31 of 34 children with APIGN, which are not able to stabilize a preformed C3 convertase (22, 23). All these observations suggest the implication of the alternative complement pathway, leading to terminal complement pathway activation, and the generation of the terminal complement complexes C5b-9 and C5a. The release of C5a increases the expression of endothelial adhesion molecules, which generates a pro-inflammatory environment and apoptosis (8). In addition; the terminal complement complex C5b-9, which is a potent pro-inflammatory complex, binds on endothelial and phagocytic cells, and causes vascular endothelium damage, resulting in endothelitis and cellular lysis. Parra et al. recently demonstrated glomerular deposits of the terminal complement complex C5b-9 in the renal biopsies of patients with poststreptococcal glomerulonephritis (9). Moreover, Matsell et al. demonstrated a significant increase of the fluid phase plasma of C5b-9 in the acute period of poststreptococcal glomerulonephritis, followed by a decrease of C5b-9 concentrations in the convalescent period (10).

Several reports also emphasize that PIGN can also be associated with an acquired (i.e., transient C3NeF, anti-Factor B, or anti-Factor H autoantibodies) or hereditary (i.e., Complement Factor H-related protein five protein deficiency) dysregulation of the alternative complement pathway (22–26). The presence of anti-factor B antibodies has been observed by Chauvet et al. in a cohort of children with APIGN (22). Chehade et al. recently reported the first case of severe PIGN with anti-factor H antibody, successfully treated with eculizumab (11). In addition, Fremeaux-Bacchi et al. reported the presence of a transient C3NeF autoantibody in three children with APIGN (26). These findings all emphasize the role of the alternative complement pathway activation in the pathophysiological mechanism of APIGN. In the first case, C3NeF autoantibody was present; however, in the second case, C3NeF autoantibody was not detected. This latter observation shows that terminal complement pathway activation might be common in PIGN, and in severe cases, eculizumab might be indicated. In the second case, and this is a limitation to our observation, an additional role of methylprednisolone cannot be ruled out. However, the patient failed to answer to that therapy, staying oliguric for 3 days.

In conclusion, we add to the current literature two children with severe APIGN and alternative complement pathway activation who responded extremely well to eculizumab. These observations emphasize the role of the alternative complement pathway activation and the benefits of C5 blockade in severe forms of APIGN. They also paved the way to clinical randomized trials. Prospective studies are needed to confirm the beneficial effect of eculizumab in severe PIGN. However, the generally favorable outcome of PIGN in the western world will render the completion of large prospective trials difficult. We recommend specific exploration of the alternative pathway, e.g., C3 and C5b-9 levels, and serologic screening for C3NeF, anti–factor B and H autoantibodies in severe cases of APIGN.

## Data Availability Statement

The original contributions presented in the study are included in the article/supplementary material, further inquiries can be directed to the corresponding author/s.

## Ethics Statement

Ethical review and approval was not required for the study on human participants in accordance with the local legislation and institutional requirements. Written informed consent to participate in this study was provided by the participants' legal guardian/next of kin.

## Author Contributions

HC and SS conceptualized and designed the study, coordinated and supervised data collection, drafted the initial manuscript, and reviewed and revised the manuscript. GG, FC, SR, GP, DT, AS, and IR contributed to the therapeutic decision and data collection, reviewed and revised the manuscript. NT-R and MP conceptualized and designed the study, coordinated and supervised data collection, and revised the manuscript. All authors approved the final manuscript as submitted and agree to be accountable for all aspects of the work.

## Conflict of Interest

The authors declare that the research was conducted in the absence of any commercial or financial relationships that could be construed as a potential conflict of interest.

## Publisher's Note

All claims expressed in this article are solely those of the authors and do not necessarily represent those of their affiliated organizations, or those of the publisher, the editors and the reviewers. Any product that may be evaluated in this article, or claim that may be made by its manufacturer, is not guaranteed or endorsed by the publisher.

## Abbreviations

APIGN: acute post-infections glomerulonephritis
C3NeF: C3 Nephritic Factor.
